# Comparison of three validated systems to analyse spinal shape and motion

**DOI:** 10.1038/s41598-022-13891-x

**Published:** 2022-06-17

**Authors:** Bettina Dreischarf, Esther Koch, Marcel Dreischarf, Hendrik Schmidt, Matthias Pumberger, Luis Becker

**Affiliations:** 1 Berlin Institute of Health, Julius Wolff Institute for Biomechanics and Musculoskeletal Regeneration, Charité-Universitätsmedizin Berlin, Augustenburger Platz 1, 13353 Berlin, Germany; 2 Center for Musculoskeletal Surgery, Charité-University Medicine Berlin, Charitéplatz 1, 10117 Berlin, Germany

**Keywords:** Anatomy, Musculoskeletal system, Diagnostic markers, Musculoskeletal system

## Abstract

The assessment of spinal shape and mobility is of great importance for long-term therapy evaluation. As frequent radiation should be avoided, especially in children, non-invasive measurements have gained increasing importance. Their comparability between each other however stays elusive. Three non-invasive measurement tools have been compared to each other: Idiag M360, raster stereography and Epionics SPINE. 30 volunteers (15 females/15 males) have each been assessed by each system, investigating lumbar lordosis, thoracic kyphosis and spinal range-of-motion in the sagittal plane. Lumbar lordosis differed significantly (p < 0.001) between measurement devices but correlated significant to each other (Pearson’s r 0.5–0.6). Regarding thoracic kyphosis no significant difference and a high correlation (r = 0.8) could be shown between Idiag M360 and raster stereography. For lumbar mobility resulting measurements differed significantly and correlated only moderate between Idiag M360 and Epionics SPINE. Although the different measurement systems are moderate to high correlated to each other, their absolute agreement is limited. This might be explained by differences in their angle definition for lordotic and kyphotic angle, their measurement placement, or their capturing of mobility (static vs. dynamic assessment). Therefore, for long-term evaluation of the back profile, inter-modal comparison of values between different non-invasive devices should be avoided.

## Introduction

As the prevalence and incidence of low back pain (LBP) constantly rise^1^, the number of clinical examinations and the costs for healthcare due to LBP are tremendously increasing^2^. In cases of chronic LBP, X-rays are often performed serially, contrary to international guidelines^3^. Especially in children and adolescents, whereas a prevalence of LBP of up to 40% is reported, the use of ionizing radiation raises ethical questions for sequential documentation^4,5^. Therefore, in recent years, alternative—radiation-free—methods have been developed to track and monitor postural deformities and thus, claim to replace at least some of the follow-up radiological measurements. These methods focus mainly on assessing lumbar spinal shape and mobility (range of motion, RoM), which are common measures in clinical examinations to determine spinal dysfunction and serve as indicators in monitoring changes of patients pre-, during and post- therapy over time^6^.

Apart from systems that are in use in sports medicine like Vicon^7^, Zebris^8^, 3D SpineMoveGuard^9^ or X-Sens sensors^10^, or those used for workplace analyses such as Lumbar Motion Monitor^11^ or CUELA system^12^, some measurement tools have been implemented in the clinical setting, for example, the Idiag M360 (MediMouse, Idiag AG, Fehraltorf, Switzerland)^13–15^, the Epionics SPINE (Epionics Medical GmbH, Potsdam, Germany)^16^ or the Formetric III raster sterography (Diers International GmbH, Schlangenbad, Germany)^17,18^. All three systems track patient’s spinal shape (lordosis and kyphosis) and mobility in the sagittal plane (RoM in flexion (RoF) and extension (RoE)) by measuring postural changes over time. However, the measuring instruments differ in several aspects. Idiag M360 measures sagittal along the processi spinose in a static position using photoelectric sensors. Raster Sterography (Formetric III) captures through a 3-dimensional image of the body surface in a static position using photoelectric sensors. Epionics SPINE measures the back shape by resistive sensors attached paravertebrally to the skin. The system is able to evaluate dynamic movements. To what extent these technical differences lead to differences in the measurement outcome has not yet been investigated. For all three systems, the literature reports moderate to good inter- and intra-rater reliability. For Idiag M360, reliability in the sagittal plane with an intra-class correlation coefficients (ICC) of 0.57 up to 0.95^15,19^ and a correlation to radiographic imaging with Spearman coefficient of r = 0.86^13^ is reported. For raster sterography, ICC ranges from 0.79 to 0.99^20–22^ with a correlation to radiographic imaging of r^2^ > 0.5^23^. For Epionics SPINE, an ICC of 0.79–0.87 is reported^16^, a direct comparison to radiographic imaging does not exist. For the clinical examination parameter fingertip to floor distance (MFTF), the literature reports an ICC reliability of up to 0.99^24^. However, even if the obtained results of some of the devices are already compared against radiographic imaging and presented good correlation, the comparability of the output data between the devices for the back shape stays elusive.

Therefore, the aim of this study was to examine the correlation and the absolute agreement of three currently used devices with a reported high reliability: the Idiag M360, the raster stereography and the Epionics SPINE system. The identification of possible measurement differences should contribute to the further development of radiation-free back measurement methods and consequently to clinical quality assurance.

## Methods

### Study participants

A total of 30 asymptomatic volunteers (15 females, 15 males) were included. The participants had no low back pain or previous spinal surgery. The mean volunteer’s age was 30.9 ± 4.6 years (females: 30.6 ± 4.0, males: 31.2 ± 5.4), mean height 174.0 ± 9.1 cm (females: 166.6 ± 5.4 cm, males: 181.4 ± 5.2 cm), mean weight 68.7 ± 12.9 kg (females: 58.9 ± 7.2 kg, males: 78.5 ± 9.4 kg) and mean body mass index (BMI) 22.5 ± 2.8 kg/m^2^ (females: 21.2 ± 2.7 kg/m^2^, males: 23.8 ± 2.2 kg/m^2^).

### Study design

All participants completed a measurement protocol that included measurements with three different devices: Idiag M360 (MediMouse, Idiag AG, Fehraltorf, Switzerland), Formetric III raster sterography (Diers International GmbH, Schlangenbad, Germany) and Epionics SPINE (Epionics Medical GmbH, Potsdam, Germany) in upright standing as well as in upper body flexion and extension (functional analyses).

In standing, all three devises allow the measurement of the lumbar lordosis (LL), whereas Idiag M360 and raster stereography further allow the assessment of the thoracic kyphosis (TK) during relaxed standing. For the functional spinal motion analysis, Idiag M360 and Epionics SPINE allow the assessment of RoF, RoE and full sagittal range of motion (RoM; sum of RoF and RoE) during maximal upper body bending.

### Measurement protocol

All three measurement-systems were employed at the same day within in protocol of approximately 90 min to exclude diurnal variations. The measurements were performed by one of the authors with an experience of 3 years with the used measurement devices. To assess intra-rater reliability, all measurements were repeated five times. The protocol started with the Epionics SPINE measurements. For this, the patients were asked to undress the upper body as well as the feet, and to stand upright with the feet shoulder-width apart and the knees extended. This was defined as the standard leg position and standardized for inter-device comparisons by using two markers on the floor for foot positioning. After that relevant landmarks were identified and the hollow plasters were attached, according to the description in the subsection Epionics SPINE. This was followed by a two-minute rest period in relaxed sitting position. The measurement with Epionics SPINE was performed in five consecutive cycles starting with neutral upright standing and standard leg position, a subsequent maximum ventral flexion with the task to touch the ground with the fingertips or hands, when even possible while holding knees fully extended. This was followed by a maximum reclination with loosely hanging arms, head reclination and with persistent knee extension ending with a return to the neutral position. These procedure where performed five times consequently. After completion of five cycles, the plasters were removed and the patient rested for five minutes in sitting position. After this rest period, the patient was asked to return to the standardized neutral position; the markings were made according to the description of the subsection Idiag M360. After marking a two-minute resting period was taken in a sitting position. Afterwards the Idiag M360 measurements were performed in upright standing, ventral flexion and dorsal extension. Patients were asked to hold end position for about 30 s for measurement. This cycle was repeated five times. The measurements with Idiag M360 were followed by a rest period of five minutes in a relaxed sitting position. This was followed by five measurements using raster sterography (Formetric III). For this, patients had to step onto a measurement platform and position himself in an upright neutral position with his knees extended, whereupon the measurements were performed. After each measurement, the subject had to step off the measurement platform and step onto the platform again for the next measurement cycle. Five measurement cycles with Raster sterography were performed. Additionally the modified fingertip to floor distance (MFTF) was assessed once 25. For inter-device and sex comparisons, the mean values of the five measurements with each device were reported and taken into account.

### Employed measurement devices

#### Idiag M360

**Figure 1:**
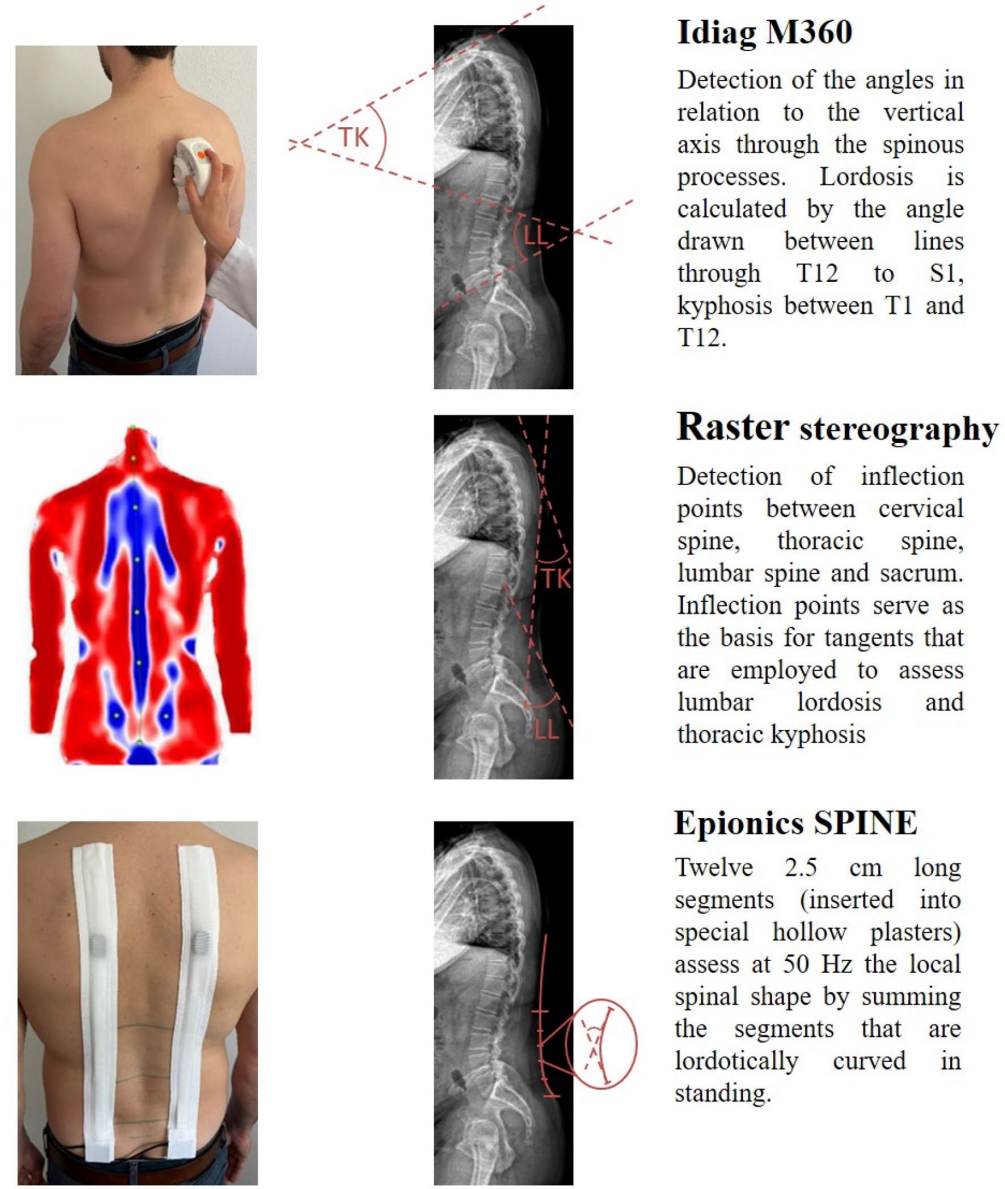
Comparison of the measurement devices and their definition for the lordosis and kyphotic angles.

The Idiag M360 is a hand-held computer-assisted electro-mechanical device that allows the assessment of the spinal shape using two rolling wheels that transfer the spinal contour via Bluetooth to a computer. For this, the tool is guided along the spine on the spinous processes starting at the C7 and ending at the caudal reference point or the top of the anal crease, respectively^15,19^. For the Idiag M360 measurements, the spinous process of C7, a reference point 2 cm below the connection line of the left and right PSIS and the top of the anal crease (approximately S3) were measured by cloth tape and marked by a skin pen. The Idiag M360 records the back length in step length of 1.3 mm. The 3D orientation of the measuring device is assigned to the specific location with a frequency of 150 Hz. By detecting the shape of each spinous processes, an imaginary line is drawn perpendicular to skin surface along each midline of the vertebrae, calculating lordotic angles between vertebrae as depicted in Fig. 1. In accordance with the measurement-guidelines, maximum upper body flexion and extension were performed with extended knees. During extension the arms were crossed in front of the body. The system’s reliability was investigated previously. A more detailed description of the system can be found elsewhere^26,27^.

#### Raster sterography (Formetric III)

The raster stereography is a non-radiological photogrammetric method that bases on the principles of triangulation. A system of light-lines is projected onto the subject’s back and subsequently distorted as a function of the three-dimensional back surface, with the aim to reconstruct the individual spinal shape^28,29^. The system is able to determine the inflection points between cervical spine, thoracic spine, lumbar spine and sacrum in the median sagittal plane as well as specific back surface landmarks such as the spinous process automatically. Therefore the spinous processus C7 is detected as the spinous process closest to the inflection point between thoracic kyphosis and cervical lordosis. These inflection points serve as the basis for tangents, which are employed to assess LL and TK as seen in Fig. 1. The system does not allow the determination of maximal range of flexion or range of extension during maximal upper body bending. In the present study, the measurement mode “3D-Average” was employed, which averages 30 measurements in 5 s to account for small posture variations. The system’s reliability in particular for sagittal plane parameters was investigated previously^20–22^ and a detailed description of the system can be found elsewhere^30,31^.

#### Epionics SPINE

The Epionics SPINE uses strain-gauge measurement technology for the detection of back shape and motion. Two flexible sensor strips that consist of each twelve 2.5 cm long segments (inserted into special hollow plasters) are placed standardized at a distance of 7.5 cm from the mid-sagittal plane with the lowest sensor segment positioned relative to the PSIS (approximately S1). Therefore, in agreement with the systems’ measurement guidelines for Epionics SPINE the left and right posterior superior iliac spines (PSIS) were marked and horizontally connected. A distance of 7.5 cm from the mid-sagittal plane was measured by cloth tape at the connecting line between the PSIS as well as 10 and 25 cm more cranial from this connecting line on each side, to which the application of the hollow plasters were adjusted. The system assesses at 50 Hz the local spinal shape in each sensor segment of 2.5 cm length, from which the lordotically curved segments in standing are individually summed up to assess the LL as shown in Fig. 1. In accordance with the measurement-guidelines, the volunteers performed maximal upper body flexion and extension keeping the knees extended. The system’s reliability was investigated previously^16^ and a more detailed description of the system can be found elsewhere^32,33^.

### Statistical analysis

The intra-rater reliability (IRR) was evaluated by ICCs for each device to quantify the degree of agreement and was evaluated following Cicchetti^34–36^. Data were tested for normal distribution using the Kolmogorov–Smirnow test. The repeated measures analysis of variance (RM-ANOVA) with Bonferroni post hoc test and sex as a between-subjects factor was used to examine the influence of the three different devices and sex for LL. To detect statistical differences in each sex in LL a RM-ANOVA were performed in male and female separately. For comparison of TK between Idiag M360 and Raster stereography as well as comparison between RoM, RoF, RoE between Idiag M360 and Epionics SPINE paired samples t-test was used. For detection of sex differences for LL and TK unpaired t-test was performed. Correlations were observed by Pearson’s correlation coefficient. A *p *value < 0.05 was considered as statistically significant.

### Ethics approval

The Charité University Berlin Ethics Board (EA1/204/16) approved this study. Each participant gave written informed consent, for publication of images informed consent was obtained. All methods were performed in accordance with the Declarations of Helsinki.

## Results

The Kolmogorov-Smirnow test demonstrated that LL, TK, RoF, RoE and RoM followed normal distribution for the entire cohort and for males and females separately.

### Intra-rater reliability analysis and comparison with reference values

The intra-rater reliability for the five repetitions for all investigated parameters in each measurement device separately demonstrated excellent reliability, with ICC greater than 0.951 (range 0.951–0.999) (Table 1).

**Table 1:** Intraclass correlation (ICC) between the five single measurements of each device for the investigated parameters.

	Lordosis (95% CI)	Kyphosis (95% CI)	RoF (95% CI)	RoE (95% CI)	RoM (95% CI)
MediMouse	0.986 (0.976–0.993)	0.986 (0.976–0.993)	0.968 (0.946–0.983)	0.951 (0.916–0.974)	0.982 (0.969–0.991)
Raster stereography	0.989 (0.982–0.994)	0.968 (0.949–0.983)	–	–	–
Epionics Spine	0.988 (0.979–0.994)	–	0.999 (0.999–1.000)	0.988 (0.979–0.994)	0.993 (0.988–0.997)

*CI* confidence interval, *RoM* range of motion, *RoF* range of flexion, *RoE* range of extension.

### Lumbar lordosis

For the entire study population as well as for males and females separately, the assessed LL differed significantly between the three employed systems, with significantly smaller values determined by the Idiag M360 compared to the two other systems (Table 2). In RM-ANOVA accounting for different measurement devices and sex as a between-subjects factor, both measurement device (p < 0.001, ηp^2^ (effect size) = 0.659) and sex (p = 0.006, ηp^2^ (effect size) = 0.239) presented significant influence on LL, however no significant interaction between sex and measurement device on LL was observed (p = 0.593, ηp^2^ (effect size) = 0.018). In agreement, all systems detected a larger lordosis in women compared to men. The absolute differences in lordosis between sexes differed between systems (Idiag M360: 8.8°; Raster stereography: 5.4°; Epionics SPINE: 6.6°) resulting in significant differences between sexes only for the Idiag M360 (p = 0.007, Cohen’s d = 1.072) and Epionics SPINE (p = 0.019, Cohen’s d = 0.906). Correlation analysis revealed a moderate correlation for the LL between systems with correlation coefficients between 0.54 and 0.61 (Table 3).

**Table 2:** Lumbar lordosis and thoracic kyphosis in upright standing for the entire study population (overall) as well as males and females separately.

	Idiag M360	Raster stereography	Epionics SPINE	RM-ANOVA	Post hoc Bonferroni		
	Mean (SD)	Mean (SD)	Mean (SD)	p value	ID vs. RS	RS vs. ES	ES vs. ID
**Lordosis (overall)**	30.1 (8.7)	40.3 (8.4)	37.9 (7.9)	** < 0.001**	** < 0.001**	0.277	** < 0.001**
Male	25.9 (9.4)	37.6 (8.9)	34.6 (8.4)	** < 0.001**	**0.001**	0.559	**0.006**
Female	34.2 (5.6)	43.0 (7.1)	41.2 (5.9)	** < 0.001**	**0.001**	0.931	**0.001**

	Idiag M360	Raster stereography		t-test			
**Kyphosis (overall)**	43.3 (9.8)	44.4 (6.5)	–	0.343	–	–	–
Male	47.2 (10.1)	47.1 (6.1)	–	0.928	–	–	–
Female	39.4 (8.1)	41.7 (5.8)	–	0.131			

Significant differences (p value < 0.05) are marked in bold. *ID* Idiag M360, *RS* Raster stereography, *ES* epionics SPINE.

**Table 3:** Correlation (Pearson) between measurement data (mean values) obtained by the different devices and modified fingertip-to-floor distance; r = correlation coefficient; MFTF = fingertip to floor distance; all correlations that are statistically significant (p value < 0.05) are marked in bold.

	Idiag M360 r (p value)	Raster stereography r (p value)	Epionics SPINE r (p value)	MFTF r (p value)
**Lordosis**				
Idiag M360	**–**	**0.54** (.002)	**0.61** (< .001)	–
Raster stereography	**0.54** (.002)	**–**	**0.56** (.001)	–
Epionics SPINE	**0.61** (< .001)	**0.56** (.002)	**–**	–
**Kyphosis**				
Idiag M360	**–**	**0.78** (< .001)	**–**	–
Raster stereography	**0.78** (< .001)	**–**	**–**	–
Epionics SPINE	**–**	**–**	**–**	–
**RoF**				
Idiag M360	**–**	**–**	**0.48** (.008)	0.05 (.815)
Raster stereography	**–**	**–**	**–**	–
Epionics SPINE	**0.48** (.008)	**–**	**–**	**0.42** (.022)
**RoE**				
Idiag M360	**–**	**–**	**0.53** (.002)	0.30 (.113)
Raster stereography	**–**	**–**	**–**	
Epionics SPINE	**0.53** (.002)	**–**	**–**	**0.54** (.002)
**RoM**				
Idiag M360	**–**	**–**	**0.47** (.009)	0.24 (.197)
Raster stereography	**–**	**–**	**–**	–
Epionics SPINE	**0.47** (.009)	**–**	**–**	**0.62** (< .001)

### Thoracic kyphosis

The assessment of the thoracic kyphosis resulted in non-significant differences between Idiag M360 and raster stereography for the entire cohort as well as for males and females separately (Table 2). Consistently in both systems, the thoracic kyphosis was significantly larger in males than in females and differed significantly between sexes as detected by both systems (p = 0.026, Cohen’s d = 0.892 for Idiag M360; p = 0.021, Cohen’s d = 0.859 for raster stereography). Correlation between both systems revealed a high and significant correlation (r = 0.78) for the assessed kyphosis values (Table 3).

### Lumbar range of flexion, range of extension, range of motion

**Figure 2:**
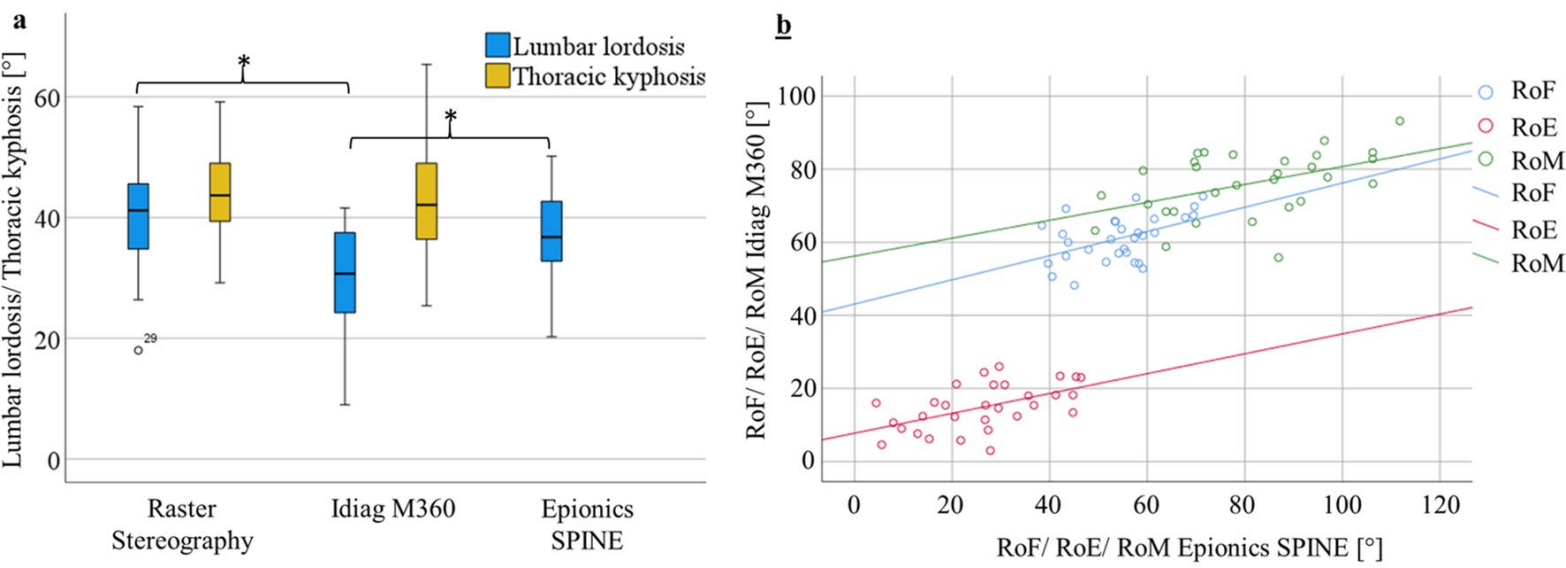
Plotted data for lumbar lordosis, thoracic kyphosis, RoF, RoE and RoM for each measurement device. (**a**) The measured lumbar lordosis (blue) and thoracic kyphosis (yellow) for each measurement device. (**b**) The range of flexion (RoF), extension (RoE) and range of motion (RoM) of the Idiag M360 and the Epionics SPINE. Significante differences are marked by asterisk.

For the entire study population, as well as for both sexes, the absolute values for RoF and RoE obtained with the Idiag M360 and Epionics Spine significantly differed from each other (Fig. 2, Table 4). The RoF was significantly larger, when obtained with the Idiag M360, whereas the RoE was significantly smaller, when measured with the Idiag M360. For the RoM, non-significant difference was obtained between systems. Consistently, both systems measured non-significant differences between males and females for the RoF (p = 0.946 Idiag M360, p = 0.288 Epionics SPINE), RoE (p = 0.523 Idiag M360, p = 0.393 Epionics SPINE) and RoM (p = 0.616 Idiag M360, p = 0.229 Epionics SPINE). For all three motion-parameters, a significant correlation between both systems was observed, ranging between r = 0.47 for the RoM to r = 0.53 for the RoE (Table 3).

**Table 4:** Descriptive statistics (mean, standard deviation) for lumbar flexion and extension for the whole study population (overall) as well as males and females separately for Idiag M360 and Epionics SPINE.

	Idiag M360	Epionics SPINE	t-test
	Mean (SD)	Mean (SD)	p value
**RoF (overall)**	61.0 (6.3)	54.1 (9.2)	** < 0.001**
Male	60.9 (7.4)	52.3 (11.1)	**0.002**
Female	61.1 (5.3)	55.9 (6.7)	**0.018**
**RoE (overall)**	14.9 (6.3)	26.4 (12.4)	** < 0.001**
Male	14.2 (7.2)	24.4 (11.5)	**0.001**
Female	15.7 (5.4)	28.4 (13.3)	**0.001**
**RoM (overall)**	76.0 (8.9)	80.5 (16.9)	0.106
Male	75.1 (9.5)	76.7 (16.8)	0.691
Female	76.8 (8.4)	84.3 (16.8)	0.063

p values base on paired t-test.

### Fingertip to floor distance (MFTF)

The modified fingertip to floor distance was also correlated to the mobility values RoF, RoE and RoM. Moderate to good correlations ranging from 0.42 to 0.62 could be detected between the MFTF distance and the Epionics SPINE results, whereas correlations between MFTF distance and Idiag M360 were poor and non-significant (Table 3).

## Discussion

The current gold standard for analysing patients’ spinal shape and mobility are radiological x-rays. As during the documentation of patients’ therapy progress, several repeated measurements are required, non-invasive measurement methods have become more and more significant, as they have the advantage of being radiation-free, enabling several measurements without any harm for the patient. However, the comparability of the measurement results of the devices against each other—even if a high intra- and interrater reliability is provided stays elusive. The present study therefore aimed to analyze three commonly used non-invasive measurement systems, to investigate their correlation to each other and to test their comparability regarding resulting angles.

The results of the present study show that in general the results of the single devices are significantly correlated. However, significant differences in total values were detected for LL, RoF, RoE and RoM between Idiag M360 and Epionics SPINE as well as for TK between Idiag M360 and raster stereography. The obtained results for the lumbar lordosis in upright standing between Idiag M360 and raster stereography differed up to 33.8%. Therefore the measured angles obtained with one of the devices could be compared to the measurements of another one in a clinical setting just to a limited extent.

The three devices differed from each other in several factors which might have led to differences between the total values. For example, the Idiag M360 measures the spinal curvature directly on the mid-sagittal plane follow the spinous processes^37^, while the Epionics SPINE system measures paravertebrally the muscle contour^16^. Moreover, the measured RoMs are not directly comparable as the Idiag M360, for example, examines flexion and extension in a static, full bended position where the subject has to hold the position for several seconds^37^. In contrast, Epionics SPINE captures movements while subjects perform the motion^16^. In addition, the devices differ in their definition of the lordotic and kyphotic angles^16,37^.

One of the three above mentioned differences might also be responsible for the fact, that the Idiag M360 and the Epionics SPINE system detect absolute differences in lordosis and kyphosis between both sexes while the raster-stereography does not account for these sex specifics. Whereas the literature reports sex differences in LL, TK, RoF, RoE and RoM^38,39^. Moreover, the Idiag M360 detected significantly higher values for the RoF and significantly lower values for RoE compared to Epionics SPINE. These differences diminished when comparing the sum of RoF and RoE (= RoM). This result emphasizes that it is important to examine not only the total range of motion, which is often used in clinical assessment as the predominant parameter of spinal function, but to also investigate the amount of flexion and extension separately.

Differences between Idiag M360 and Epionics SPINE become also very obvious when correlating both measurements to the commonly used MFTF distance, which is a current clinical orientating parameter for assessing patients’ flexibility^24^. Measurements of Epionics SPINE correlate quite well to MFTF, while those of Idiag M360 do not, emphasizing again differences in the measurement technique or the definition of the measured angle. However, the MFTF is dependent not only on trunk flexion but also on hip flexion and body proportions such as arm, hand and trunk length^25^. The obtained measurements for LL, flexion and extension by the Idiag M360^13,15,19^ as by Epionics SPINE^20^ were within the range of those reported in the literature. The results for thoracic kyphosis and LL in upright standing from raster stereography are also in line with the results reported for that device^40^.

The measurement devices presented an excellent ICC of 0.951–0.999 in our study. While in the literature for the Idiag M360 an ICC of 0.57 up to 0.95 is reported^15,19^, in our study an ICC of 0.951–0.986 was obtained. For the raster stereography the literature reports an ICC of 0.79–0.99 while we examined values of 0.968–0.989^20–22^. For the Epionics SPINE, we obtained an ICC of 0.993–0.999 while the literature reports values of 0.79–0.87^16^. The excellent ICC shown in our study compared to the literature could possibly have resulted from the execution in our study by a single investigator experienced with each of the measurement devices, the close time interval of our measurements with persistent skin markers (Idiag M360) or the remaining of the measurement instrument on the body between measurement cycles (Epionics SPINE) as well as the standardization of the body position between the measurements cycles of each measurement device. Additionally, the literature reports a strong relationship to radiographic measurements for the Idiag M360 as well as for raster stereography. Despite the good reliability the literature reports a strong relationship to radiographic imaging for Idiag M360^13,14^ as well as for raster stereography^23^.

The presented study had several limitations. To keep the influence of soft tissue on the measurement results low, only subjects were included who had a BMI < 27. The measurement differences between the systems, would possibly increase at higher BMIs, as Epionics SPINE measures directly on soft tissue and is thus stronger BMI influenced, while the Idiag M360 measures on the spinous processes and is thus lower BMI influenced. Furthermore, the order of measurements could not be randomized because, due to regular patient examinations, we had limited access to raster stereography. Age may also have an effect on the differences in measurement, as skin aging, and increased skin movability may have affected the procedures. We performed our analysis on patients without known spinal pathology, structural abnormalities or back pain, which might result in limited transferability to the evaluation of patients with low back pain. The measurements in our study were performed by a single rater with repeated measurements. Despite the high ICC for intra- and inter-rater reliability reported in the literature, this may limit the transferability of our study to repeated diagnostics by different investigators. Although a good reliability was shown for each single measurement devise, differences in the measurement outcome can be caused by the different measurement techniques when using several devices on one patient making therapy progress hard to evaluate or falsify the therapy effects altogether.

It can be concluded that the three used measurement devices with the advantage over X-ray not to expose the subjects to radiation differ to some extent in their exact outcome. Therefore, the interpretation and comparability of the results stays challenging. A significant correlation of total values against radiological imaging is described for both Idiag M360 and raster stereography measurements, however, analyses of the total statistical agreement between radiographic imaging and non-radiographic measurement tools are lacking^13,23^. Accordingly, the non-radiological measurement instruments should be used less as an instrument for the primary detection of pathologies but X-ray measurements can be supplemented by one of the three described methods, which, due to their high reliability, may reduce the frequency of radiographic follow-up examinations. The choice, which measurement method should be used, depends on the individual indication for the follow-up examinations. The Idiag M360 and raster stereography are static measurements that are well suited for monitoring the back profile and scoliosis progression. The Idiag M360 can also be used to assess the RoM. Epionics SPINE allow statements to be made about the long- and short-term back functionality as well as the back profile in daily activities. Due to the dynamic measurement, the movement sequence can be described precisely. However, for long-term evaluation of the back profile, inter-modal comparison of values between different non-invasive devices should be avoided.
